# Supplementary figures and images for: Signal-sensing triggers the shutdown of HemKR, regulating heme and iron metabolism in the spirochete Leptospira biflexa
Source: PLoS One. 2024 Sep 26;19(9):e0311040. doi: 10.1371/journal.pone.0311040 (PMC11426443; doi:10.1371/journal.pone.0311040)

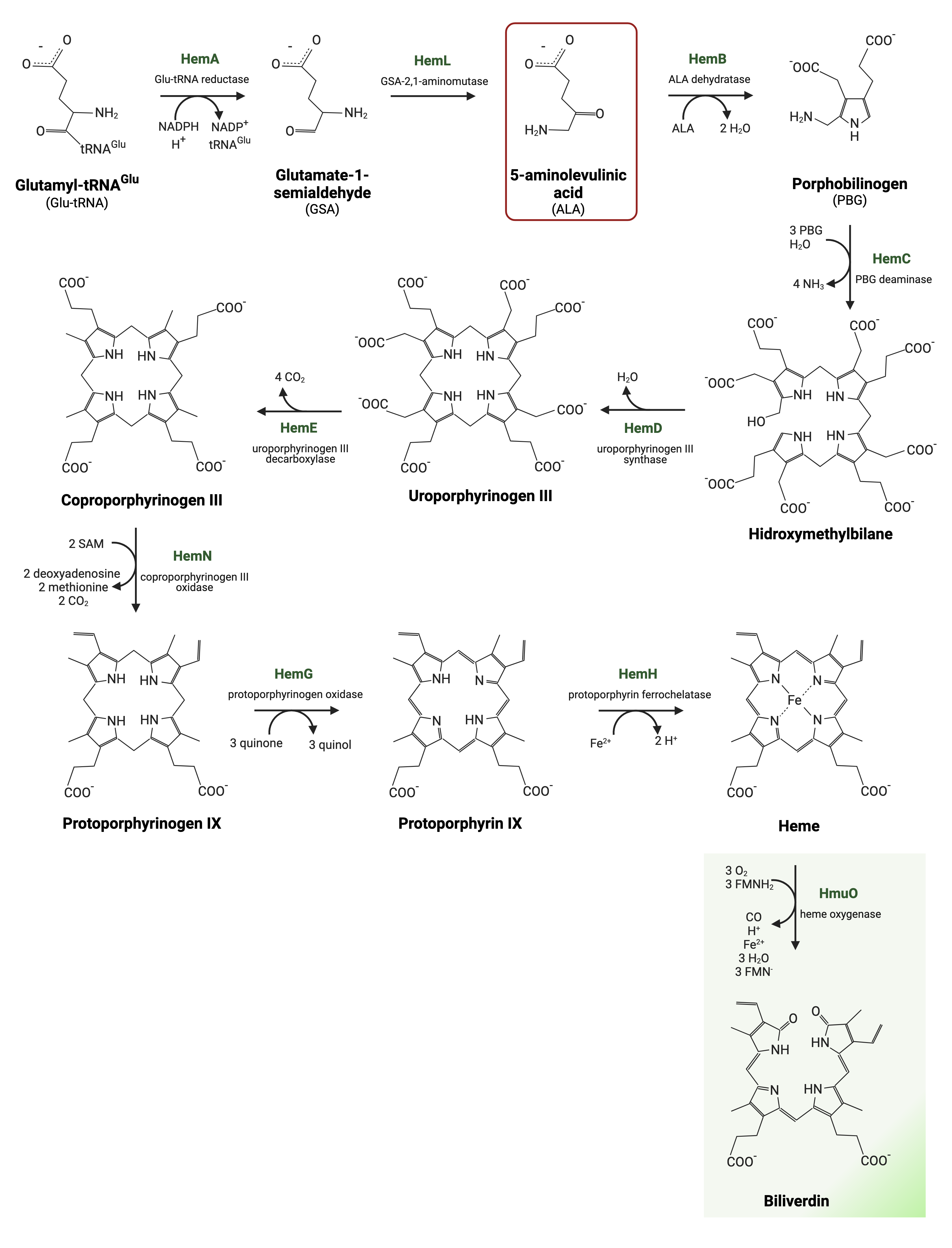

Supplement: S1 Fig — As in many other bacteria, Leptospira spp. possess all the genes coding for anabolic enzymes of the so called C5 pathway for de novo heme biosynthesis. Starting from glutamyl-tRNAGlu, as initial precursor, the committed substrate 5- aminolevulinic acid (ALA) is generated (boxed in red). Four ALA moieties are needed to build the tetrapyrrole backbone of porphyrins including heme. Note that HemC and HemD are found within a single bifunctional enzyme in Leptospira spp [15]. The first step of heme degradation, catalyzed by heme oxygenase, is highlighted at the end of the pathway. (TIF) [file pone.0311040.s001.tif]

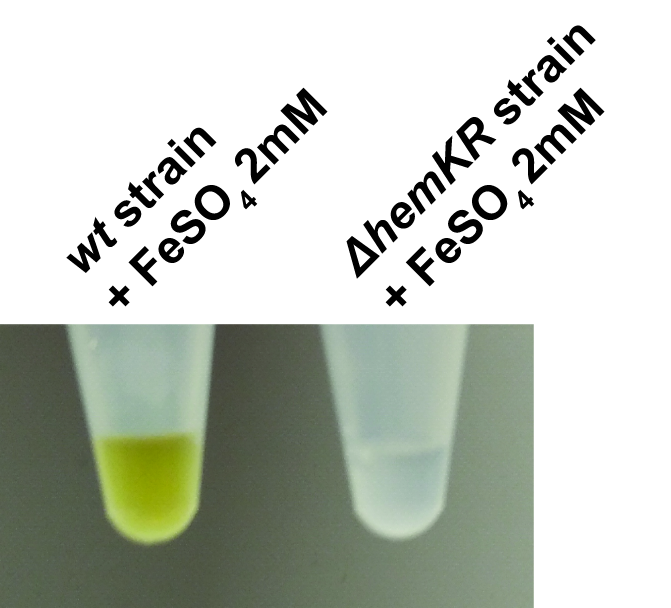

Supplement: S2 Fig — Samples, as labeled, were submitted to standard RNA extraction procedures in preparation for whole mRNA transcriptomic sequencing. For unknown reasons, the wt strain–and not the ΔhemKR KO–exhibited an abnormal behavior if previously treated with excess iron: an insoluble yellow precipitate was formed during RNA extraction, which impaired RNA recovery prior to sequencing. (TIF) [file pone.0311040.s002.tif]
