# Supplementary material for: Signal-sensing triggers the shutdown of HemKR, regulating heme and iron metabolism in the spirochete Leptospira biflexa
Source: PLoS One. 2024 Sep 26;19(9):e0311040. doi: 10.1371/journal.pone.0311040 (PMC11426443; doi:10.1371/journal.pone.0311040)
Supplement: S3 Table — (DOCX) [file pone.0311040.s005.docx]

**S3 Table. Selected genes differentially expressed when *wt* *L. biflexa* cells are exposed to 5-aminolevulinic acid**

| **Genomic locus** | **Annotated gene name** | **FC**^¶^ | **P value**^§^ | **Additional comments** |
| --- | --- | --- | --- | --- |
| LEPBamiscRNA3 | -- | 23.55 | 0.0061 | non-coding RNA Flavo-1 (Rfam-predicted |
| LEPBIa1691 | -- | 6.56 | 0.0194 | hypothetical |
| LEPBIa2499 | *ppc* | 4.88 | 0.0004 | phosphoenolpyruvate carboxylase |
| LEPBIa3373 | -- | 3.98 | 0.0058 | hypothetical |
| LEPBIa1173 | -- | 3.80 | 1.32e-7 | hypothetical |
| LEPBIa0669 | *hmuO* | 1.91 | 4.22e-32 | heme oxygenase involved in heme degradation (Wilks, 2002; Murray et al., 2009) |
| LEPBIa3270 | -- | 1.88 | 2.40e-20 | hypothetical (putative anti-anti-sigma factor) |
| LEPBIa2792 | -- | 0.17 | 1.11e-15 | hypothetical |
| LEPBIa3240 |  | 0.20 | 1.56e-11 | hypothetical |
| LEPBIa1171 | *hemA* | 0.29 | 1.83e-60 | Glu-tRNA reductase (catalyses the first step of porphyrin biosynthesis) / first gene of the operon *hemACBLENG* involved in heme-biosynthesis, all found down-regulated |
| LEPBIa3432 | -- | 0.33 | 0.0298 | TonB-dependent outer-membrane receptor/transporter homologous to PhuR |
| pLEPBI0018 | -- | 0.34 | 2.85e-5 | putative TonB-dependent PhuR-like receptor (neighbour to the *hemTUVS* locus) |
| pLEPBI0015 | *hemT* | 0.36 | 0.0001 | periplasmic heme-carrier protein (HemT) / First gene of the operon *hemTUVS* (which also encodes the inner-membrane heme-permease complex ABC-transporter HemUV; and the cytoplasmic heme-sequestering protein HemS) |
| LEPBIa0149 | *exbB1* | 0.53 | 0.0002 | part of the TonB-dependent system, supplies energy to drive iron/siderophore/porphyrin outer-membrane transporters / It is the first gene of the operon *exbB1/exbD1* coding for the TonB-inner membrane complex |

**¶** FC = Fold-change (ratio of each gene’s transcription level comparing ALA-treated *vs* untreated cells) / over-expressed genes are highlighted in red and under-expressed ones, in blue.

**§** P value = raw p-value from statistical test (probability of the null hypothesis that the expression of treated and untreated are equal), adjusted according to the Benjamini-Hochber (Benjamini & Hochberg, 1995) multiple testing model, considering false discovery rate. Lower than 0.05 is considered significant.
