# Supplementary material for: Signal-sensing triggers the shutdown of HemKR, regulating heme and iron metabolism in the spirochete Leptospira biflexa
Source: PLoS One. 2024 Sep 26;19(9):e0311040. doi: 10.1371/journal.pone.0311040 (PMC11426443; doi:10.1371/journal.pone.0311040)
Supplement: S5 Table — (DOCX) [file pone.0311040.s007.docx]

**S5 Table. Selected genes differentially expressed when *wt* *L. biflexa* cells are exposed to 2,2’-dipyridyl (iron deficit)**

| **Genomic locus** | **Annotated gene name** | **FC^¶^** | **P value^§^** | **Additional comments** |
| --- | --- | --- | --- | --- |
| LEPBIa2760 | -- | 730.30 | 2.5e-131 | PhuR-like TonB-dependent outer-membrane receptor/transporter ; reported as desferrioxamine receptor, sensitive to iron depletion (Louvel et al., 2006); comprised within an upregulated operon that includes a putative iron-dependent membrane protein (LEPBIa2762) |
| pLEPBI0015 | *hemT* | 132.20 | 1.9e-100 | periplasmic heme-carrier protein HemT; comprised within the *hemTUVS* operon that includes inner-membrane heme-permease complex HemUV and cytoplasmic heme-sequestering protein HemS |
| pLEPBI0018 | -- | 66.68 | 8.8e-73 | hypothetical (putative TonB-dependent outer membrane receptor) |
| LEPBIa1883 | *fecA* | 66.07 | 6.98e-98 | outer-membrane TonB-dependent ferric citrate receptor/transporter FecA |
| LEPBIa0390 | -- | 53.59 | 1.1e-247 | hypothetical (putative long-chain fatty acid transport protein) |
| LEPBIa0669 | *hmuO* | 20.85 | 1e-50 | heme oxygenase (involved in heme degradation) |
| LEPBIa1691 | -- | 6.16 | 0.0245 | Hypothetical |
| LEPBIa0249 | -- | 3.54 | 3.3e-22 | Hypothetical |
| LEPBIa2210 | -- | 0.14 | 0.0110 | hypothetical |
| LEPBIa2180 | -- | 0.14 | 6.36e-20 | hypothetical |
| LEPBIa1794 | *bfrB* | 0.28 | 1.6e-115 | bacterioferritin (iron storage) |
| LEPBIb0063 | -- | 0.32 | 1.06e-47 | nitric oxidoreductase (comprising a heme-binding cytochrome C-like domain) |
| LEPBIa1171 | *hemA* | 0.35 | 1.07e-44 | Glu-tRNA reductase (catalyses the first step of porphyrin biosynthesis) / It is the first gene of the operon *hemACBLENG* involved in heme-biosynthesis |
| LEPBIa0149 | *exbB1* | 0.45 | 2.37e-6 | inner-membrane importer subcomplex (together with co-regulated ExbD1 encoded in the same operon), part of the TonB-dependent system that supplies energy to drive iron/siderophore/porphyrin outer-membrane transporters |

**¶** FC = Fold-change (ratio of each gene’s transcription level comparing ALA-treated *vs* untreated cells) / overexpressed genes are highlighted in red, underexpressed ones in blue.

**§** P value = raw p-value from statistical test (probability of the null hypothesis that the expression of treated and untreated are equal), adjusted according to the Benjamini-Hochber (Benjamini & Hochberg, 1995) multiple testing model, considering false discovery rate. Lower than 0.05 is considered significant (highlighted in bold fonts).
