# Supplementary material for: Signal-sensing triggers the shutdown of HemKR, regulating heme and iron metabolism in the spirochete Leptospira biflexa
Source: PLoS One. 2024 Sep 26;19(9):e0311040. doi: 10.1371/journal.pone.0311040 (PMC11426443; doi:10.1371/journal.pone.0311040)
Supplement: S1 Raw images — (PDF) [file pone.0311040.s008.pdf]

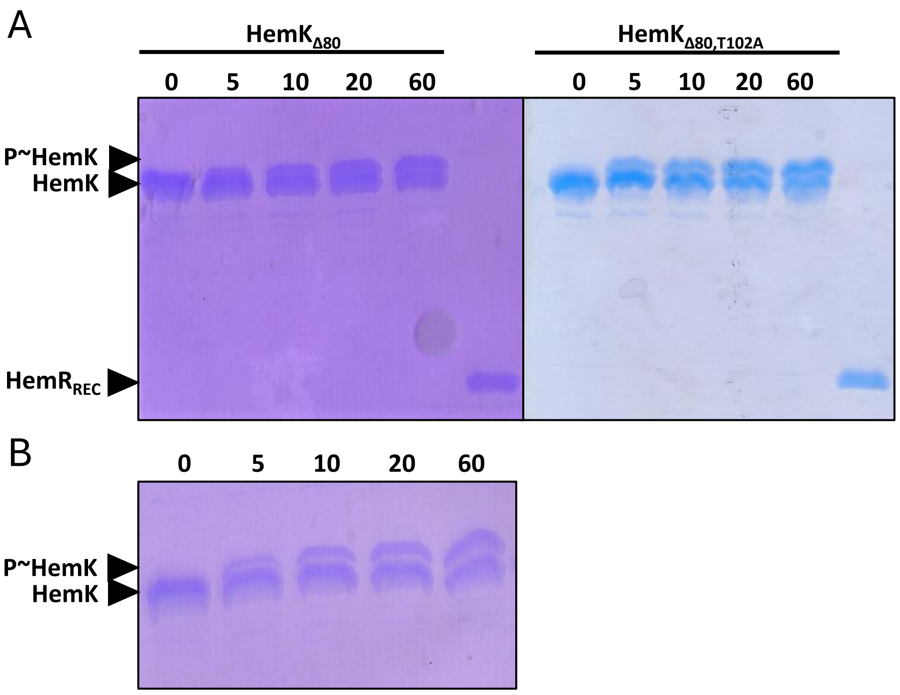

Raw Image 1. (A) Autophosphorylation of $HemK_{\Delta80}$ (left panel) and the phosphatase-null point-mutant $HemK_{\Delta80,T102A}$ (right), incubated for indicated times with 5mM ATP-Mg^{2+}. $HemR_{REC}$ (with molecular weight ~15 kDa) is only shown as a reference. (B) PhosTag-SDS-PAGE shown in Figure 1A, improved from experiment shown above in A (left panel). Note that phosphorylated $HemK_{\Delta80}$ (P~HemK) is better separated from non-phosphorylated $HemK_{\Delta80}$.

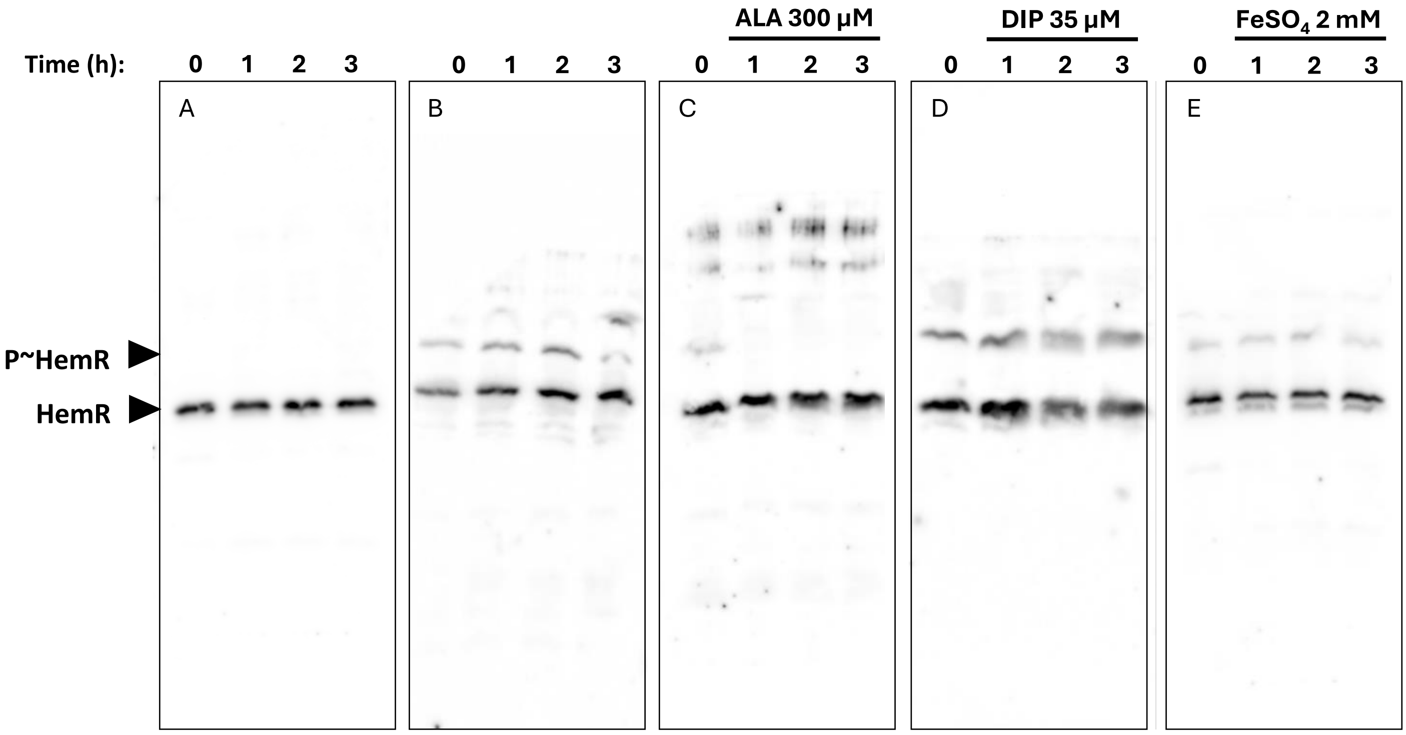

Raw Image 2. PhosTag-SDS-PAGE of *L. biflexa wt* whole protein extracts, followed by Western blot using an anti-HemR antibody, as shown in Figure 2A. Panel A: no PhosTag added. Panels B, C, D & E: PhosTag added to polyacrylamide gels.

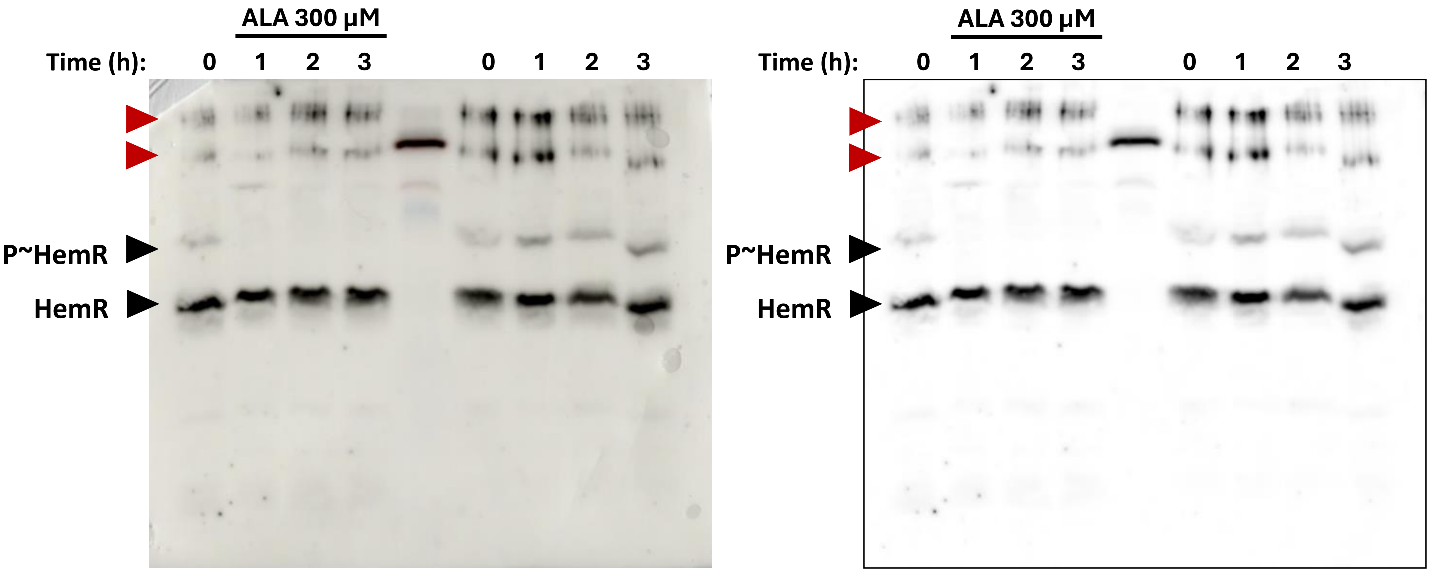

Raw Image 3. PhosTag-SDS-PAGE of *L. biflexa wt* whole protein extracts, followed by Western blot using an anti-HemR antibody, as shown in Figure 2A. Note that the nonspecific bands (red arrows) shown in Raw Figure 1, panel C appear in the lanes corresponding to ALA-treated, as well as in the lanes corresponding to untreated bacteria.

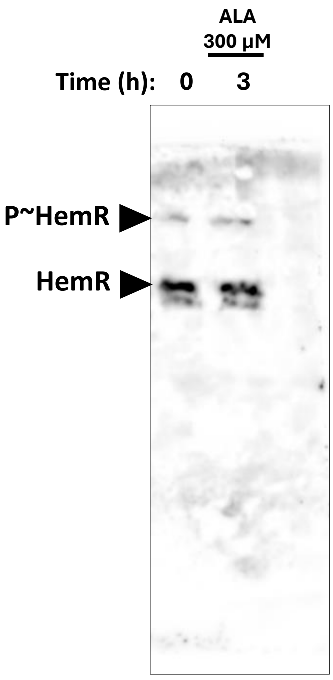

Raw Image 4. PhosTag-SDS-PAGE of whole protein extracts of *L. biflexa* Δ*hemKR* strain complemented with P$hemK_{T102A}$/*hemR* expression plasmid, followed by Western blot using an anti-HemR antibody, as shown in Figure 2C.
