# Supplementary material for: Signal-sensing triggers the shutdown of HemKR, regulating heme and iron metabolism in the spirochete Leptospira biflexa
Source: PLoS One. 2024 Sep 26;19(9):e0311040. doi: 10.1371/journal.pone.0311040 (PMC11426443; doi:10.1371/journal.pone.0311040)
Supplement: Attachment — Submitted filename: response2reviewers.docx [file pone.0311040.s009.docx]

Reviewers' comments:

**Reviewer #1:** 
*This is a well written manuscript. They investigated that how environmental signal sensing triggers the shutdown of HemKR, a two-component system (TCS) and regulates the homeostasis of Iron and heme metabolism in a saprophytic spirochete Leptospira biflexa.

The study is well designed, well executed and moreover results are well discussed. But the study is limited to a non-pathogenic leptospiral strain which lack the virulence factors associated with pathogenic Leptospira sp. Since the study aimed at exploring the influence of iron/heme metabolism and their response receptors (RR) and TCS target genes essential in regulating the heme homeostasis of Leptospira during infection. The authors claimed 5-aminolevulinic acid (ALA), a porphyrin precursor triggers the shutdown of HemKR pathway by inducing the HemK phosphatase activity towards the HemR. HemR dephosphorylation further leads to differential expression of many genes important in heme metabolism and the inactivation of HemR also generates iron-deficit tolerant phenotype.

For this purpose, they utilized wildtype L. biflexa and generated a double knockout mutant of L. biflexa, ΔhemKR. The approach of engineering the saprophytic strain easier than the virulent strain and it is popular in the field. The study needs few modifications to improve the quality of the manuscript. Below are few recommendations and concerns*

We thank Dr Suman Kundu for his time and positive feedback.

*1. It would be interesting to see the mechanism in a virulent strain such as L. interrogans. The authors mentioned that the L. biflexa is similar to pathogenic Leptospira species which raises concern as they may look similar but have different membrane proteins which may yield different outcomes during heme metabolism and infection process.*

We agree with Dr Kundu in that it will indeed be very interesting to study this HemKR two-component system in a pathogenic *Leptospira* species.

However, to perform a similar study with *L. interrogans*, goes beyond the scope of this paper at this point. It is definitely something that we are pursuing. We agree with the reviewer that HemKR might work differently in both species, and/or could be distinctively regulated, even though their high identity strongly suggests that this TCS will share at least some similar physiologic roles.

In any case, as suggested also by Dr Stevenson (PloS ONE Editor of this manuscript), we have carefully checked and edited our ms, in order to avoid any misleading, oversimplistic extrapolation from the saprophyte model to pathogenic species.

*2. The author referred few articles to introduce the heme/iron sensors found across the Leptospira sp. that includes L. interrogans. More recently, Orillard et al in 2022, provided some clear insight about the unusual membrane bound PAS-heme oxygen sensor of L. interrogans. This could be a potential question and use of a pathogenic strain therefore is recommended.*

Thank you for pointing this out. We have now extended the Discussion so that the referred work by Orillard et al can be commented in context. These new sentences also allow us to put in perspective the interesting work by N Benaroudj (Inst Pasteur), who has been showing how the PerR type of regulators sense reactive species of oxygen allowing *L. interrogans* to adapt to oxidative stress (see further elaboration about this in point 4 below).

*3. The shuttle vector pMaORI is of great choice for this type of study and it creates avenue for many other research questions targeting saprophytic L. biflexa. Is the shuttle vector equally manipulative for pathogenic Leptospira?*

The short answer is yes, this shuttle plasmid can be used to transform *L. interrogans* (as well as *L. biflexa*). The reference we cite in the ms (Pappas et al, 2015) describes this in detail.

However, and following up on point 1 above, even though repeating our HemKR studies on a pathogenic species such as *L. interrogans* will be extremely interesting, it does require a very laborious, focused investigation that clearly goes beyond the scope of our work now. Obtaining knockouts by homologous recombination (e.g. the Δ*hemKR^-^* double KO) is still much harder compared to the saprophyte model.

*4. The author provided evidence that mutant ΔhemKR strain of L. biflexa are more tolerant when there is an iron deficit condition. What is the role of hemKR TCS in the oxidative stress response?*

This is an extremely interesting question. We have not studied the oxidative stress response, which indeed in several other cases has been shown to be linked in some specific way to iron/heme homeostasis. Further research will be able to respond to this aspect.

We have now highlighted the article by Zavala-Alvarado *et al* (doi:10.1371/journal.ppat.1008904) in our Discussion. That paper shows that the transcription of the entire heme biosynthesis operon (which in pathogenic *Leptospira* species includes *hemK/hemR*) is up-regulated when *L. interrogans* is exposed to sublethal concentrations of H_2_O_2_, in a PerR-independent way. More direct evidence is required to uncover the potential relationship of HemKR-signaling and oxidative stress response, both in pathogenic as well as in saprophytic *Leptospira* species.  *5. What is the role of hemKR TCS in scavenging heme from hemoglobin? This mutant ΔhemKR model may provide better answer and that could be a potential target considering their role during pathogenesis and heme homeostasis.*

As mentioned already above, we are working to generate the Δ*hemKR^-^* KO in *L. interrogans*, so that this first study can be properly furthered in the pathogen model.

*6. The authors observed a comparatively less significant differences in transcriptional activation and/or down regulation between the wildtype and the mutant strain of L. biflexa and that aligns with the iron deficit response. Although the transcriptomic data during iron overabundance is missing due to some difficulties in RNA purification. May be that would answer whether transcriptional regulation rely on the environmental iron or not.*

Future work will likely enable us to solve the iron-excess technical problems to purify mRNA (which intriguingly, only happens with the *wt* strain and not with the Δ*hemKR^-^* KO). Perhaps working with pathogenic species could eventually be helpful, whereas also allowing us to address the potential role of this TCS during mammal infection. *7. Furthermore, it is not clear from the present study how HemKR responds to ALA and correspond to the environmental iron deficit condition. And, If it does so through transcriptional regulations, how that can influence the iron metabolism during pathogenesis process followed by a virulent Leptospira infection.*

We’re not sure whether we fully understand the question here.

HemKR responds to ALA through transcriptional regulation, indeed. Besides the direct and extensive evidence we present here (and see also the answer to point 8 below), our previous work (Morero et al 2014 Mol Microbiol) proved how phosphorylated HemR binds to the *hem*-box sequence within the promoter regions of two target loci. All evidence considered, the kinase HemK is able to sense ALA (and/or other downstream metabolites of the porphyrin biosynthesis pathway) triggering the shutdown of HemR phosphorylation.

The connection to iron homeostasis is here uncovered, yet more work is needed to fully describe the cross-talk.

Finally, with respect to the pathogen, here we only lay down a working hypothesis (that we are now pursuing as a rationale for a separate publication): when the HemKR pathway is shutdown, *L. biflexa* cells display a clear phenotype of higher tolerance to environmental iron deficit. This hypothesis must be tested experimentally in that upcoming work focused on the pathogen. *8. The author claimed that HemK possesses phosphatase activity in vivo and proposed ALA could be a potential source during iron deficit condition during leptospiral infection in the host. No in vivo results were incorporated to support this.*

That HemK possesses specific phosphatase activity towards P~HemR, is indeed proved in this paper. The prediction about the pathogen is only laid down as a hypothesis, within the Discussion section, as a perspective line to be followed up in future studies. Given the high similarity of these TCS proteins in both models, we believe this is a reasonable hypothesis to put forward, awaiting experimental confirmation. *This study provides a clear insight about the use of a saprophytic leptospiral strain which further creates opportunities for others to address different questions.*

Thank you again to Dr Suman Kundu for his valuable comments.

**Reviewer #2:**

OVERALL IMPRESSION:
*The manuscript entitled “Signal-sensing triggers the shutdown of HemKR, regulating heme and iron metabolism in the spirochete Leptospira biflexa” explores the signaling pathway of the HemKR TCS system that controls heme metabolism and import. To do so, a variety of in vitro approaches were used to carefully answer the questions about the triggering signal of HemK, its effect on activation of its phosphatase activity, the regulatory responses concerning P~HemR and HemR, and, finally, the phenotypical assays that showed that the absence of HemKR control conferred iron-deficit tolerance to leptospires.

The article has the potential to deepen the knowledge on leptospiral regulatory pathways, and metabolism. However, before its publication, the manuscript should be revised to correct a few inaccuracies and to provide additional improvements.*
We thank reviewer #2 for his/her positive feedback overall.

MAJOR ISSUES:

*1) On page 16, lines 361-362, it is written: “Transcriptomic analyses confirmed that transcription of hemK and hemR genes was indeed abolished in the mutant strain”. However, a careful examination of Table S2 shows this information is inaccurate. hemR indeed displayed much smaller numbers of read counts when we compare the ∆hemKR strain with the wt, which might indicate a severe impairment in transcription.

However, the two count tables show hemK normalized read counts in the wt and the ∆hemKR strains. Furthermore, when we evaluate the DGE tables, there is differential expression of hemK in ∆hemKR strains: in ALA-treated cultures, hemK was more expressed in comparison to untreated cultures (Log2FC = 0,551; p adj = 1,05E-09). Therefore, if we detect differential expression of hemK, after ALA treatment of ∆hemKR strains, hemK transcription is not abolished in ∆hemKR strains.

I suggest that the authors adapt the aforementioned sentence to better describe the transcriptomic data, since transcription of hemK gene was not abolished.*
We understand the reviewer’s concern, yet we did describe this in detail in the Materials and methods section (lines 230-233 of the first version manuscript):

“As for *hemK* (*LEPBIa1423*), standard differential gene expression (DGE) analyses did not detect a significant shift with respect to the *wt*; yet, a more detailed mapping on the genomic loci revealed that the reads corresponding to *hemK*, comprise only the initial 5’ fragment of the gene, upstream of the kanamycin-resistance cassette.”

Regretting this was not clear enough, we have now added an explicit note to this important detail within the main text of Results.

For the sake of this rebuttal discussion with the reviewers, let us attach a figure illustrating the actual mapping of transcriptomic reads in this particular region of the genome (Fig revision 1):

**Fig. revision 1.** Coverage of reads (in grey) mapped on the *Leptospira biflexa* reference genome. The x-axis spans the range of nucleotides (nt) 1,484,010 – 1,486,351 [according to chromosome 1 numbering of the reference *L. biflexa* sv Patoc Paris strain; depicted at the bottom of the figure using the name Leptospira_biflexa_mage.gff].

The region illustrated thus comprises genes *LEPBIa1423/hemK* (nt 1,485,464 – 1,486,351) and *LEPBIa1422/hemR* (nt 1,484,784 – 1,485,467), according to the .gff annotation file. Please note that these genes’ ORFs sit on the antisense strand, so everything must be read from right to left: the 5’end of *hemK* corresponds to nt 1,486,351; its 3’ end is nt 1,485,464; etc).

The three examples shown here correspond to three independent experiments, readily highlighting the accuracy of the point where transcription ceases (nt 1,486,065 which coincides with the kanamycin-resistance cassette insertion site), to then resume after the inserted cassette.

All reads mapping to *hemK*, correspond to the first 286 bp of the gene, explaining why standard differential gene expression (DGE) analyses did not detect a significant shift with respect to the *wt*, yet ensuring that *hemK* was effectively knocked-out (*i.e.* no HemK protein can be produced).

*2) On page 19, lines 417-421, the authors describe a new binding motif of HemR in the upstream region of LEPBIa3430/LEPBIa3431. How was this new motif identified? It is important to include the methods used to detect this binding motif, whether by in vitro or in silico analysis.*
It was identified *in silico* by searching the *hem*-box consensus sequence on the promoter regions of up-/down-regulated genes. We used the FIMO Motif Search Tool v. 5.5.1 (https://meme-suite.org/meme/tools/fimo) to perform the search. We have now added this information in the main text Results and Materials and methods sections.

MINOR ISSUES:

*1) Considering the functional analysis of recombinant TCS proteins, the transcriptomic data of wt and ∆hemKR strains, the binding motif identification, and the phenotypic evaluation of mutant and wt strains under DIP treatment, the authors proposed a signaling and regulatory model of heme metabolism in L. biflexa. I suggest the authors create a schematic figure summarizing the signaling and regulatory pathway of HemKR TCS upon ALA detection, including activated and repressed target genes. Such figure would benefit the readers and highlight your findings.*

Thank you for the suggestion: we have incorporated a schematic illustration, summarizing the main findings of our work. This corresponds to new Figure 6, within the Discussion section. Of note, the figure also allows the readers to readily identify open questions that will be addressed in future reports.

*2) The authors emphasized more than once the hypothetical importance of HemKR TCS system under iron-limiting conditions during host infection by pathogenic Leptospira. Although I agree this is an accurate interpretation of biological evidence, it would be very interesting if the authors could also interpret and discuss their data considering the importance of HemKR system under environmental conditions in water and/or soil. Not only because Leptospira biflexa is a saprophytic species, but because many pathogenic Leptospira spp. present a free-living phase on the environment in their complex life cycle, which enables them to endure between hosts. This would be a very thought-provoking addition.*
We fully agree in that this perspective would be very thought-provoking, and it certainly involves a physiologically relevant lifestyle, the free-living form, which is key for pathogenic leptospires to disseminate.

We do not have enough experimental evidence as yet, to elaborate this discussion without too much speculation. Having said this, and following the reviewer’s suggestion, we have introduced a short fragment within the Discussion section to take this facet into account.

OTHER POINTS:

*Line 55: endoflagella is spelled without a hyphen.*

Now corrected

*Line 58: “including the causative agent of leptospirosis” should be changed to “aetiological agent of leptospirosis”. A disease is multifactorial and caused by more than its infectious agent, the concentration of bacterial inoculum and the host’s immune system are also unequivocally involved. Therefore, it is not accurate to refer to pathogenic Leptospira as the causative agent of the disease.*

Now corrected, thank you for the explanation. “Etiologic agents” (in plural) seems adequate, since we are referring to the several different *Leptospira* pathogenic species.
